# High throughput deep sequencing reveals the important roles of microRNAs during sweetpotato storage at chilling temperature

**DOI:** 10.1038/s41598-017-16871-8

**Published:** 2017-11-29

**Authors:** Zeyi Xie, Aiming Wang, Hongmin Li, Jingjing Yu, Jiaojiao Jiang, Zhonghou Tang, Daifu Ma, Baohong Zhang, Yonghua Han, Zongyun Li

**Affiliations:** 1 Institute of Integrative Plant Biology, School of Life Science, Jiangsu Normal University, Xuzhou, China; 2 Jiangsu Key Laboratory of Phylogenomics and Comparative Genomics, Jiangsu Normal University, Xuzhou, China; 3 Xuzhou Institute of Agricultural Sciences in Xuhuai Distrct, Jiangsu Xuzhou Sweetpotato Research Center, Sweetpotato Research Institute, CAAS, Xuzhou, China; 4 Key Laboratory of Biology and Genetic Improvement of Sweetpotato, Ministry of Agriculture, Xuzhou, China; 5 Department of Biology, East Carolina University, Greenville, NC USA

## Abstract

Sweetpotato (*Impomoea batatas L*.) is a globally important economic food crop with a potential of becoming a bioenergy and pharmaceutical crop. Thus, studying the molecular mechanism of tuberous root development and storage is very important. However, not too much progress has been made in this field. In this study, we employed the next generation high-throughput deep sequencing technology to sequence all small RNAs and degradome of sweetpotato for systematically investigating sweetpotato response to chilling stress during storage. A total of 190 known microRNAs (miRNAs) and 191 novel miRNAs were identified, and 428 transcripts were targeted by 184 identified miRNAs. More importantly, we identified 26 miRNAs differentially expressed between chilling stress and control conditions. The expression of these miRNAs and their targets was also confirmed by qRT-PCR. Integrated analysis of small RNAs and degradome sequencing reveals that miRNA-mediated SA signaling, ABA-dependent, and ROS response pathways are involved in sweetpotato root response to chilling stress during storage.

## Introduction

Sweetpotato (*Ipomoea Batatas* Lam.) is a hexaploid (2n = 6x = 90) dicot in the *Convolvulaceae* family. Due to its rich nutritional characteristics, unfluctuating high yields, and worldwide adaptability, it is used as nourishment, animal feed, and a raw material for biofuel production. With origin in the tropics, sweetpotato is susceptible to chilling damage when stored at low temperatures. Chilling damage may occur in many different situations, including on-farm storage and in consumer refrigerators; it also happens in wholesale and retail storage facilities and in supermarket display racks as well as during transportation. Long-term exposure to low temperature results in a series of physiological changes, including vacuolar membrane degradation, mitochondrial membrane swelling, and an increased content of phenolic compounds in sweetpotato roots^1–3^. In China, about 1/3 of sweetpotato yield is affected by low-temperature storage stress, and further reducing yield and utilization. Thus identifying the factors responsible in this stress response would have a significant economic impact. In the past, although people attempted to solve this problem, the majority of studies focused on optimizing storage conditions; there are also several reports on the physiological and molecular changes in sweetpotato during cold storage stress. However, the regulatory mechanism is unclear.

MicroRNAs (miRNAs), a type of widespread non-coding small endogenous RNA ranging from approximately 20 to 24 nucleotides in length, are a crucial negative regulator in the post-transcriptional gene regulation, which regulate gene expression by targeting mRNAs for translational repression and/or guiding degradation of their mRNA targets^4^. miRNAs are widely existed throughout the entire plant kingdom and are highly conserved from plant species to species, such as from mosses to higher flowering plants, including both monocots and dicots^5^. Numerous reports show that miRNAs are involved in plant growth and development as well as response to different abiotic stresses, including extreme temperatures, drought, salinity and even nutrient deprivation as well as heavy metals^6^. Several miRNAs, including miR156, miR172 and miR398, have been identified to be responsive to chilling stress in *Prunus persica* ^7^, *Camellia sinensis* ^8^ *Glycine max* ^9^, *Solanum habrochaites* ^10^, *Solanum lycopersicum* ^11^, and *Manihot esculenta* ^12^. However, no any reports on miRNA response to chilling stress in sweet potato.

Based on a limited study, a couple of sweetpotato miRNAs and their targets have been characterized using computational and sequencing approaches. miR160, miR164 and miR166 are involved in sweetpotato root development, which have higher expression levels in fibrous roots and storage roots than that in petal, stylus and stamen^13^. Bian *et al*. identified 24 conserved miRNAs and 16 novel miRNAs by deep sequencing, and the predicted target genes included transcription factors, methyltransferase and dihydroflavonol 4-reductase^14^. Sweetpotato miRNA- targeted gene pathways may have the similar functions as in *A.thaliana* ^15^. miR156- Selenium-binding protein (SBP) regulates the early flower development and the vegetative phases, and miR168-ARGONAUTE1 improves resilience measures of the plants during viral infections. However, a connection has not yet been established between miRNAs and chilling stress in sweetpotato during storage. In previous studies, the prediction of miRNA target genes in sweetpotato was limited by *in silico* sequence alignments. In contrast, degradome sequencing can validate the large-scale discovery of miRNA target genes. Here, we combined sRNAome and degradome sequencing to identify both conserved and novel miRNAs, their targets, and expression patterns in sweetpotato under chilling stress. The identification of comprehensive sets of miRNAs and their target genes is a critical step to understanding the regulatory mechanisms or networks during chilling stress in sweetpotato.

## Results

### Physiological responses of sweetpotato under chilling stress during storage

**Figure 1:**
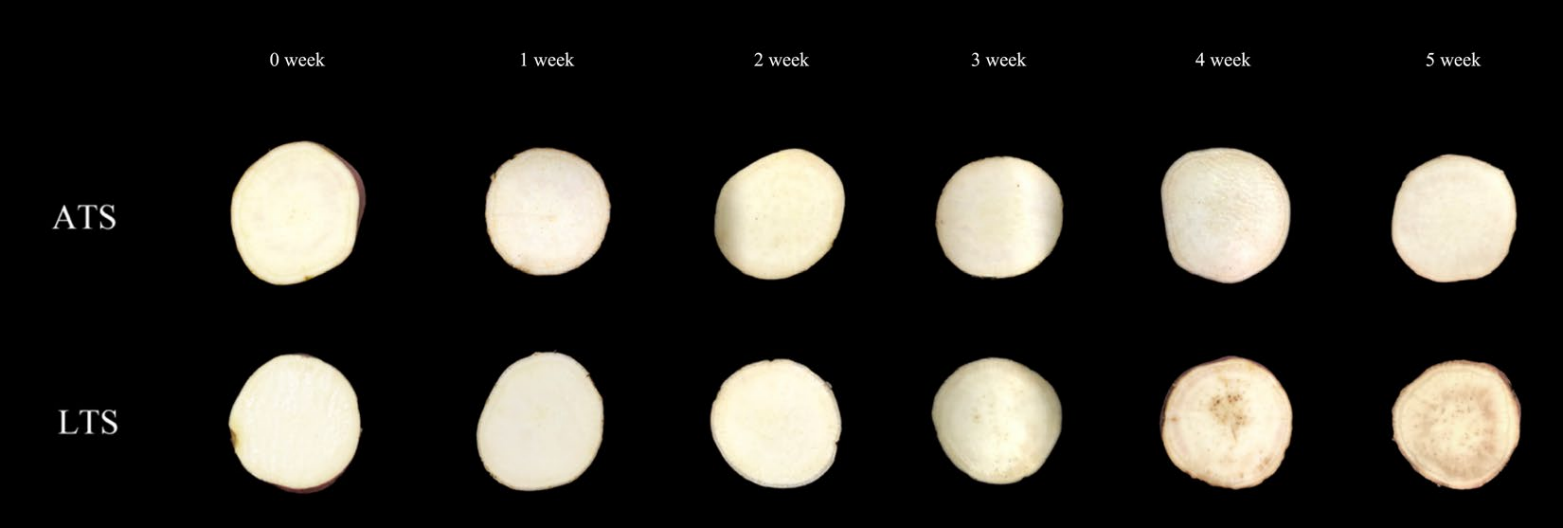
Morphological changes during storage at 4 °C (low temperature storage, LTS) or at 14 °C (appropriate temperature storage, ATS).

**Figure 2:**
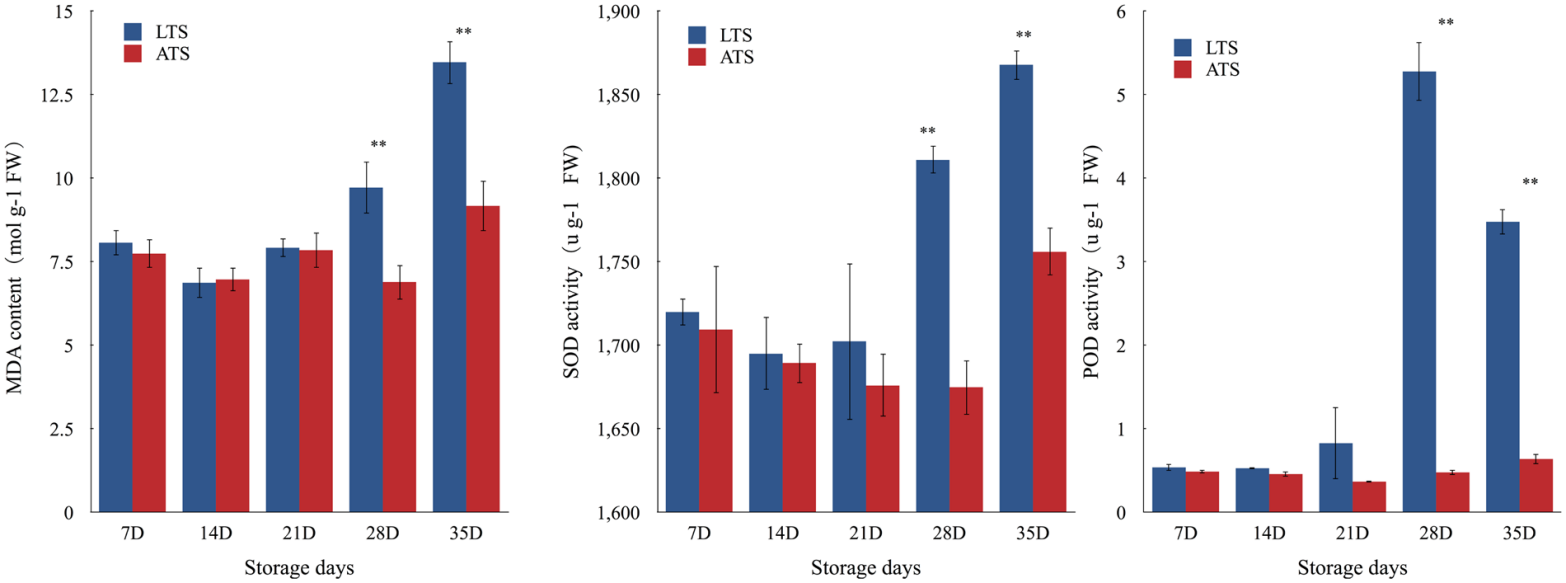
SOD activity, POD activity and MDA content in sweetpotato under chilling stress during storage. An analysis of variance (ANOVA) was employed to analyze all data. The data are presented as the mean ± SD. *p < 0.05, **p < 0.01.

Sweetpotato tuberous roots were stored at 4 °C (low temperature storage, LTS) or at 14 °C (appropriate temperature storage, ATS) for 5 weeks, respectively. Darkening of internal tissues were observed in LTS tubers at 4 weeks, while there were no significant differences in ATS (Fig. 1). To investigate the physiological responses of sweetpotato to chilling stress, we measured the activities of several enzymes, including Superoxide Dismutase (SOD), peroxidase (POD), and malondialdehyde (MDA)^16^ for three biological replicates of tuberous roots at different temperatures every week. SOD activity, POD activity and MDA content were significantly different between LTS and ATS after 4 weeks of treatments (Fig. 2). The quality of sweetpotato was also affected after 4 weeks of chilling stress. Therefore, 4 weeks of chilling treatment were selected for this study.

### Deep sequencing of sRNAome in sweetpotato

**Figure 3:**
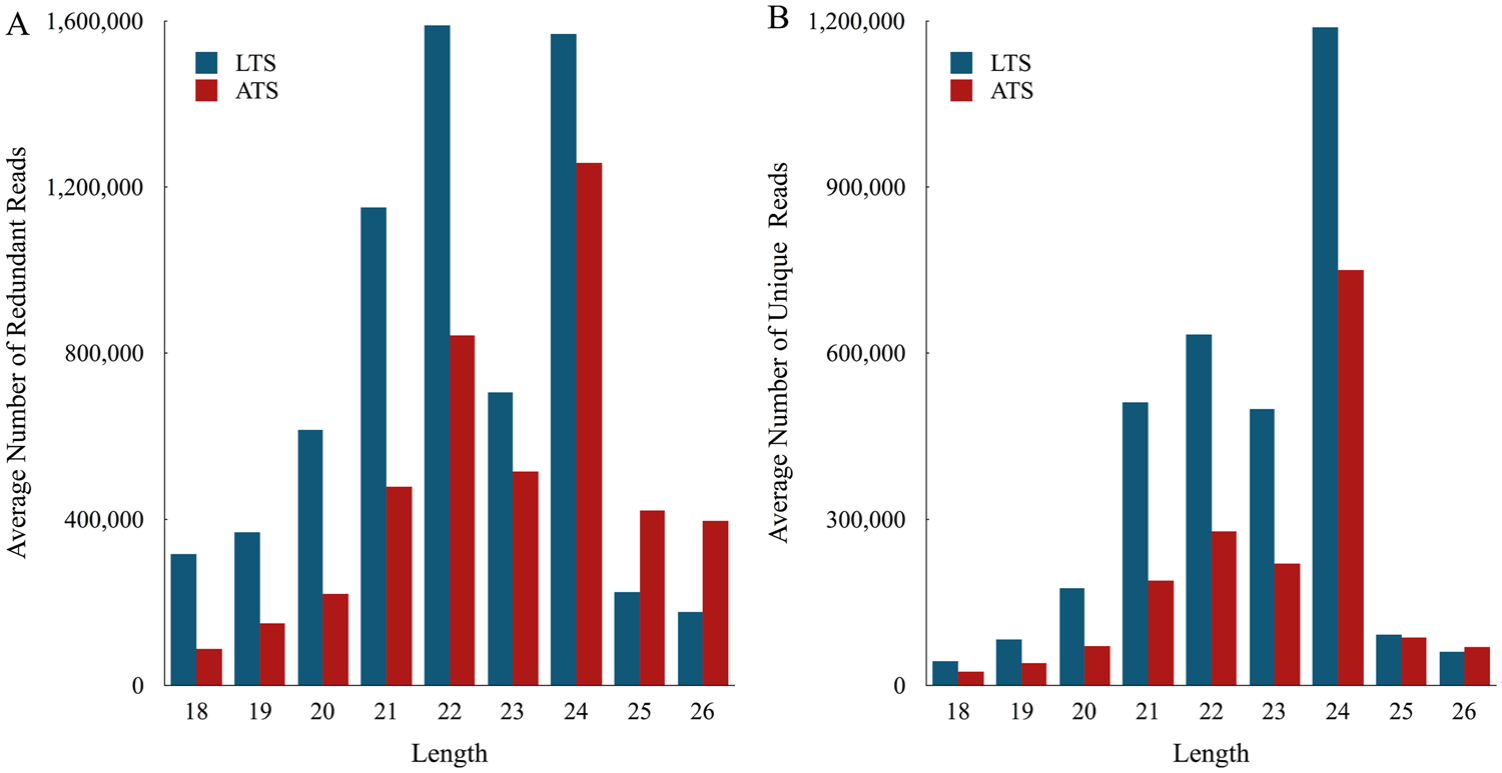
Sequence length distribution of small RNA in low temperature storage and appropriate temperature storage sweetpotato.

Single-end deep sequencing was performed by using an Illumina Hiseq. 2500 in the LC-BIO (Hangzhou, China) following the vendor’s recommended protocol for small RNAs. Data processing occurred as described previously^9^ by LC Sciences Service. An average of 11,374,096 and 11,885,609 raw sequences were generated from LTS and ATS libraries, respectively. After removal of low quality and adapter sequences, there were an average of 6,713,561 and 4,361,997 clean reads from LTS and ATS libraries, respectively (Table S1). Small RNAs with 24 and 22 nt in length were the majority of the redundant reads (Fig. 3A), and the length distribution of the unique sequences showed that 24 nt RNAs were the most abundant sequences (Fig. 3B), which accounted for approximately 36.19% and 43.45% of LTS and ATS libraries, respectively (Fig. 3B). Our result is similar to results reported in a previous study in sweetpotato^14^.

### Identification of known and novel miRNAs in sweetpotato

**Figure 4:**
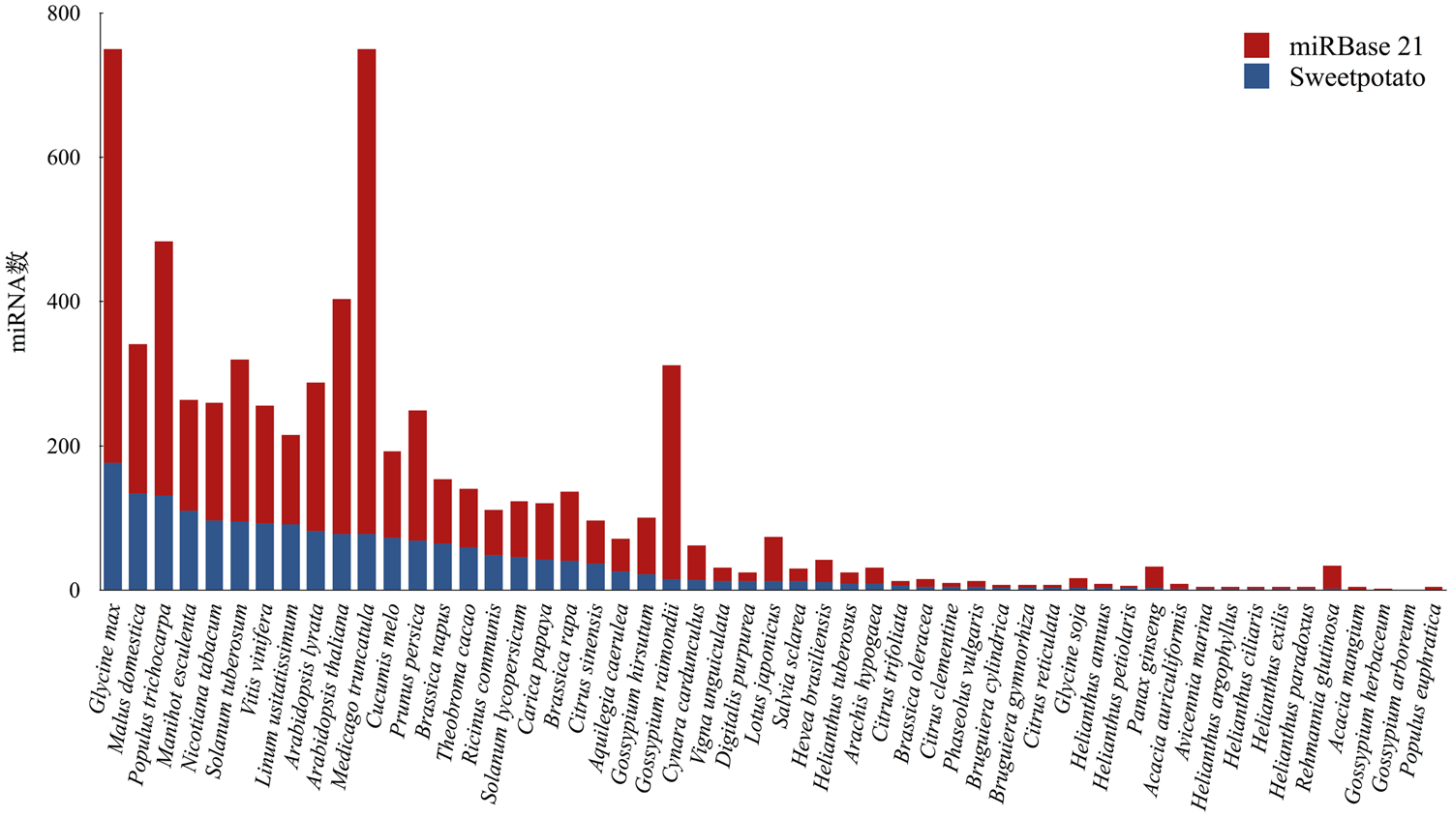
Conservation of the identified miRNA with other species in sweetpotato. The X axis is the count of miRNAs, the Y axis represents the name of the species in miRBase21. Red bar plot showing the miRNA number of the species in miRBase21, blue bar plot showing the miRNA number identified both in sweetpotato and in the species in comparison.

To identify known miRNAs in sweetpotato, all sequenced small RNA sequences were aligned to all miRNAs in the public available database, miRBase (version 21, June 2014)^17^. Based on the sequence similarity, a total of 190 mature miRNAs were identified, which represented 56 miRNA families (Table S2, Fig. S1A). It is well known that conserved miRNAs play important functions in plant growth and development as well as response to environmental stress^4,6^. After performing a homologues research in a wide range of plant species, we found that many conserved miRNA families, including miR156, miR166, miR172, miR395, miR169, and miR167 are also existed in sweetpotato (Table S3, Fig. 4). Due to the lower numbers of conserved miRNAs identified in *Manihot esculenta* and *Solanum tuberosum* compared to *Glycine max*, the highest quantity of conserved miRNAs in sweetpotato were mapped to *Glycine max* (29.67%). More closely related species, such as *Manihot esculenta* (69.93%) and *Solanum tuberosum* (41.96%) shared lower numbers of conserved miRNAs with sweetpotato. The majority of identified known sweetpotato miRNAs (44.81%) were 21 nt in length, similar to other species^18,19^. The miRNAs had a very broad range of expression, ranging from thousands of reads to fewer than 10 (Table S3). The majority of these miRNAs, such as miR396, miR319, miR482, miR384, miR858, miR164, miR168 and miR479, were relatively highly expressed in each library. For example, miR396 had an average of 13,277 and 4,595 reads in LTS and ATS libraries, respectively, and was the most abundant miRNA in both libraries. However, some miRNA families had relatively low expression (less than 10), such as miR157, miR5304, miR824, and miR8155; all of them only had a few of read number (Table S4).

To identify new miRNAs in sweetpotato, we first identified the potential miRNA precursors in Kazusa Sweet Potato GARDEN database^20^ and then predicted their secondary structures. A total of 191 putative novel miRNA candidates were identified from 183 unigenes (Fig. S1B, Table S3). The negative folding free energies of these pre-miRNA hairpin structures ranged from −174.80 to −19.70 kcal mol^−1^ with an average of ~−74.36 kcal mol^−1^, and minimal folding free energy index (MFEI)^21^ ranging from 0.90 to 2.50 with an average of 1.27. The expression of these newly identified miRNAs varied from thousands of reads to fewer than 10 reads (Table S4).

### Target validation for sweetpotato miRNAs

To better understand the regulatory function of miRNAs, computational program psRNATarget (http://plantgrn.noble.org/psRNATarget/) was employed to identify miRNA targets. After a careful search, a total of 2321 transcripts were identified to be targeted by 334 miRNAs. Conserved miRNAs targeted an average of 12.19 target genes, while the number of target genes targeted by novel miRNAs was an average of 8.45. Therefore, a transcriptome-wide experimental method is needed to directly detect cleaved miRNA targets, and this method will not rely on predictions or overexpression to show that miRNAs can regulate target gene expression in multiple signaling pathways.

High-throughput degradome sequencing was employed to identify target sites by a potential miRNA, based on the homology of miRNA and mRNA sequences from the previous transcriptome sequencing. A total of 116,587,458 raw reads were obtained with 19,501,453 (16.7%) unique reads, corresponding to 82,875 (75.3%) transcripts matching 10,310,828 (52.9%) unique reads (Table S5). After processing and analysis with CleaveLand 3.0, we identified a total of 428 transcripts targeted by 184 miRNAs. 326 and 114 transcripts were targeted 43 known miRNA families and 46 novel miRNAs, respectively. There were 189 pairs including 141 transcripts targeted by 94 miRNAs that were unique in the ATS library, while 159 pairs were unique to the LTS library, including 126 transcripts targeted by 79 miRNAs (Fig. S2, Table S6).

Similar to previous studies in sweetpotato^14,15^ and other plant species^9–11^, miRNAs were predicted to cleave more than two targets. In this study, miR156a-5p was predicted to splice 16 targets belonging to SPB-like (SPL) transcription factors, probable magnesium transporter NIPA2 and transmembrane 9 superfamily member 4. At the same time, we also observed that a same target can be regulated by a couple of miRNAs. For instance, miR156, miR157, miR159, miR319 targeted comp48166_c2 that probably encodes SBP1.

### Identification of chilling stress-responsive miRNAs and their targets in sweetpotato

Through comparing the reading number of deep sequencing between LTS and ATS libraries, it is possible for us to identify miRNAs with differential expression levels in response to chilling stress. In this study, a total of 19 known miRNAs and 7 novel miRNAs were significantly differentially expressed between LTS and ATS libraries (Table S7). 8 and 18 miRNAs showed significant down- and up-regulation in response to chilling stress, respectively.

Beside miRNAs, we also investigated miRNA targets for better understanding the potential roles of the miRNAs with different expression levels (Table 1). The target genes play a wide range of biological functions; based on their targets, the differential expressed miRNAs were classified into two groups. The first category included Iba-miR156, Iba-miR166a, Iba-miR169, Iba-miR172 and Iba-miR403, which targeted different transcription factors, including SPLs, Homeobox-leucine zipper proteins (ATHB), Nuclear factor Y(NF-Y), APETALA2/ethylene-responsive transcription factor RAP2-7 and Zinc finger protein (ZAT) transcription factors. This category appears to be involved in gene expression regulation and signal transduction. The second category of miRNAs included miRN1, miR166d, miR172 and miR403, and their targets play roles in adaptive responses in plant growth and development, such as kinase-, helicase- and transferase-coding genes.

**Table 1:** Differentially expressed miRNAs identified in LTS compared to ATS.

Index	SmallRNA	SmallRNA_seq	LTS	ATS	log2(LTS/ATS)	Transcript Annotation
1	PC-130-5p	TGAAAGTTGGATAGGATGGCC	3.75	0.00	—	MAA3, SEN1
2	Iba-miR156a-5p_L + 1-aly	TTGACAGAAGATAGAGAGCACT	2.45	0.00	—	SPL1, SPL2, SPL3, SPL12, SPL15
3	Iba-miR169g_1ss7AG-mes	CAGCCAGGGATGACTTGCCGA	7.20	0.34	4.42	SN2, NFYA1, NFYA3
4	Iba-miR166a-3p_1ss21CA-stu	TCGGACCAGGCTTCATTCCCA	23.68	2.39	3.31	ATHB-14, ATHB-15
5	Iba-miR166a-3p_1ss17TC-stu	TCGGACCAGGCTTCATCCCCC	14.58	2.73	2.42	ATHB-14, ATHB-15
6	Iba-miR166a-3p-stu	TCGGACCAGGCTTCATTCCCC	5224.33	1420.58	1.88	ATHB-14, ATHB-15
7	Iba-miR166a-3p_L + 1R-1_2-stu	TTCGGACCAGGCTTCATTCCC	22.09	3.43	2.69	ATHB-14, ATHB-15
8	Iba-miR166a-3p_L + 1-stu	TTCGGACCAGGCTTCATTCCCC	41.67	13.72	1.60	ATHB-14, ATHB-15
9	Iba-miR166d_2ss4TG16TC-gra	TGAGGGGAATGTTGTCTGGCT	77.32	18.08	2.18	SD1-8 (Precursor), PERK5, TaCab1
10	Iba-miR172a-3p_L + 1R-1-stu	GAGAATCTTGATGATGCTGCA	24.11	8.14	1.57	RAP2-7, AP2, CRK14, SD1-6 (Precursor), XRN4
11	Iba-miR403a_R-1_1ss19TC-mes	TTAGATTCACGCACAAACCC	59.19	26.55	1.16	ZAT12, MAA3, SPL12

*Log2 ratio of normalized miRNA expression in LTS compared with ATS.

**Figure 5:**
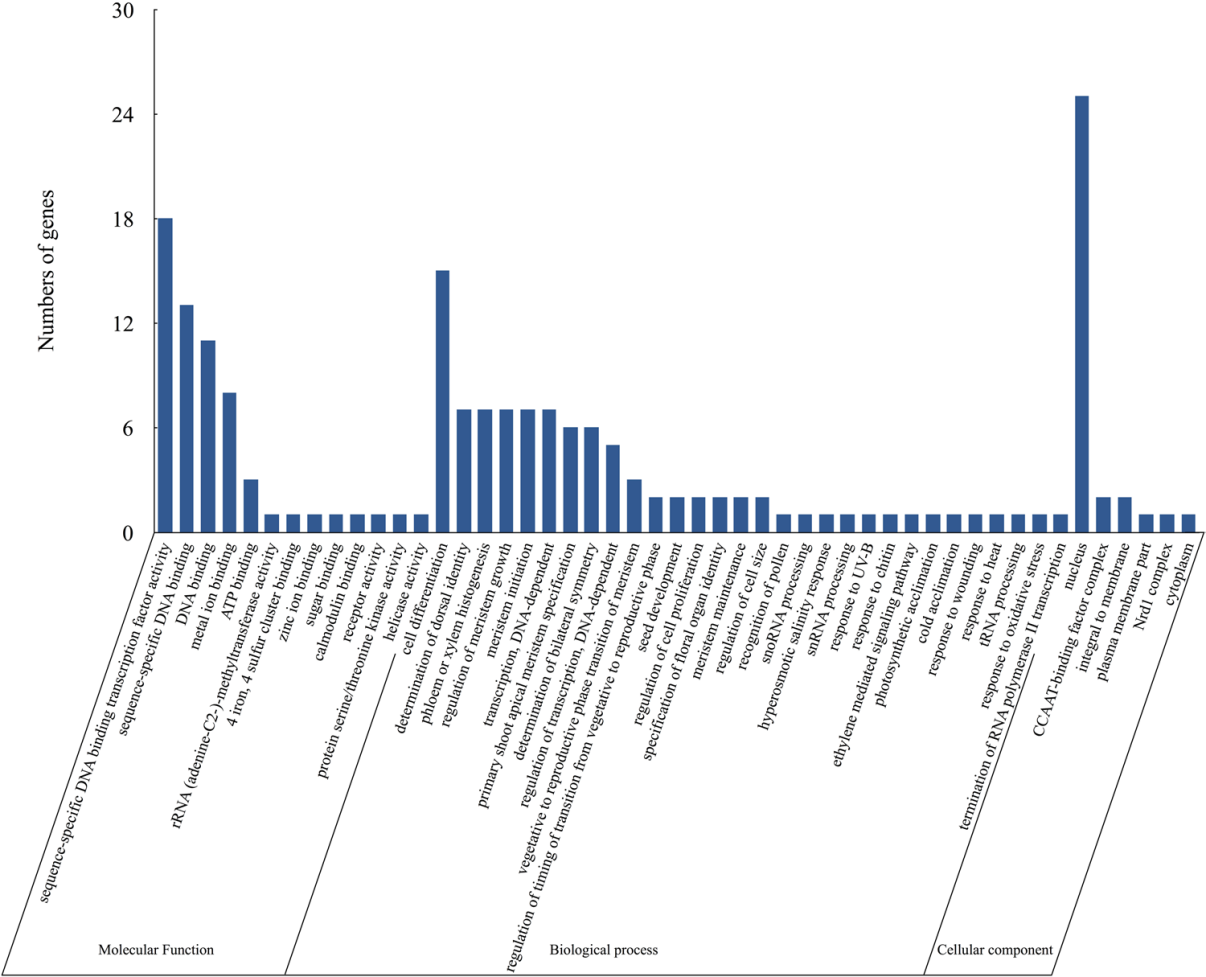
GO analysis of miRNA target genes in sweetpotato. Only the predicted target genes for miRNAs responding to chilling stress were considered.

We also performed a gene ontology (GO) analysis of the targeted genes for further understanding the miRNA functions. The target genes of the differentially expressed miRNAs were classified into 49 categories: 13 molecular functions, 30 biological processes, and 6 cell component categories. Within the molecular function category, significant enrichment was found for sequence-specific DNA binding transcription factor activity, sequence-specific DNA binding, DNA binding, metal ion binding, and ATP binding. In the biological process category, more than 50% of the targets were involved in a broad range of biological processes, including cell differentiation, meristem initiation, phloem or xylem histogenesis, determination of dorsal identity, regulation of meristem growth, and DNA-dependent transcription. In the cell component category, the most enrichment was for the nucleus (Fig. 5).

### Validation of identified miRNAs and targets in sweetpotato

**Figure 6:**
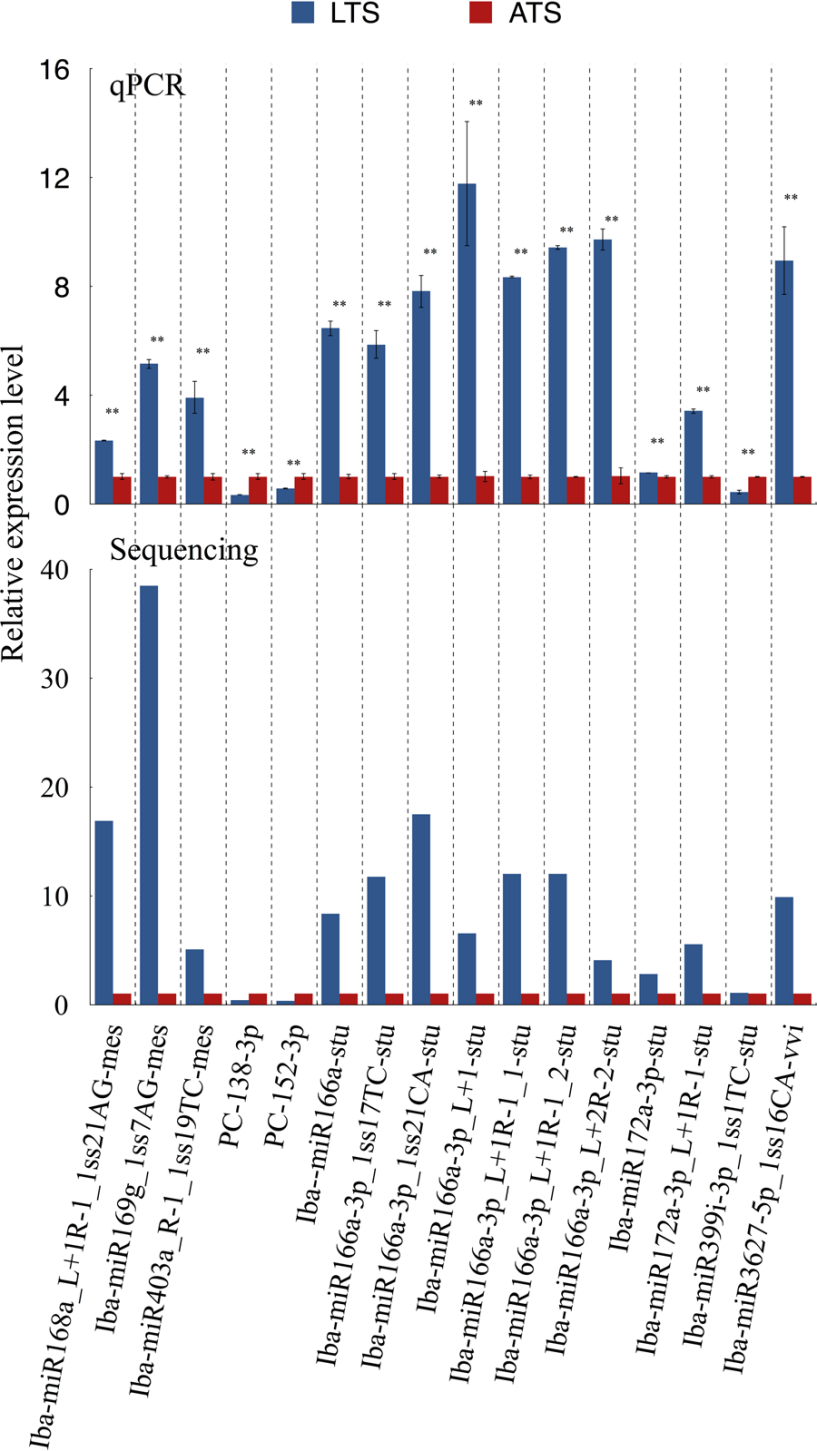
Expression patterns of these selected miRNAs obtained by qRT-PCR and sequencing. Expression of miRNAs was normalized by the level of *U6* in qRT-PCR respectively. Three biological replicates were performed for each miRNA and each treatment. An analysis of variance (ANOVA) was employed to analyze all data. The data are presented as the mean ± SD. *p < 0.05, **p < 0.01.

To validate the results generated from deep sequencing technology, we employed quantitative real time PCR (qRT-PCR) to confirm the expression of miRNAs and their targets. We randomly selected 16 differentially expressed miRNAs (including 14 known miRNAs and 2 novel miRNAs) between LTS and ATS libraries for qRT-PCR analysis. Generally speaking, the expression of the selected miRNAs were consistent between qRT-PCR and sequencing (Fig. 6). A linear regression analysis [(Sequencing value) = a (qRT-PCR value) + b] found a positive correlation between the sequencing data and the qRT-PCR, with correlation coefficients of 0.8788 (Fig. S3). The qRT-PCR expression were similar to and supported the results generated by miRNA sequencing.

**Figure 7:**
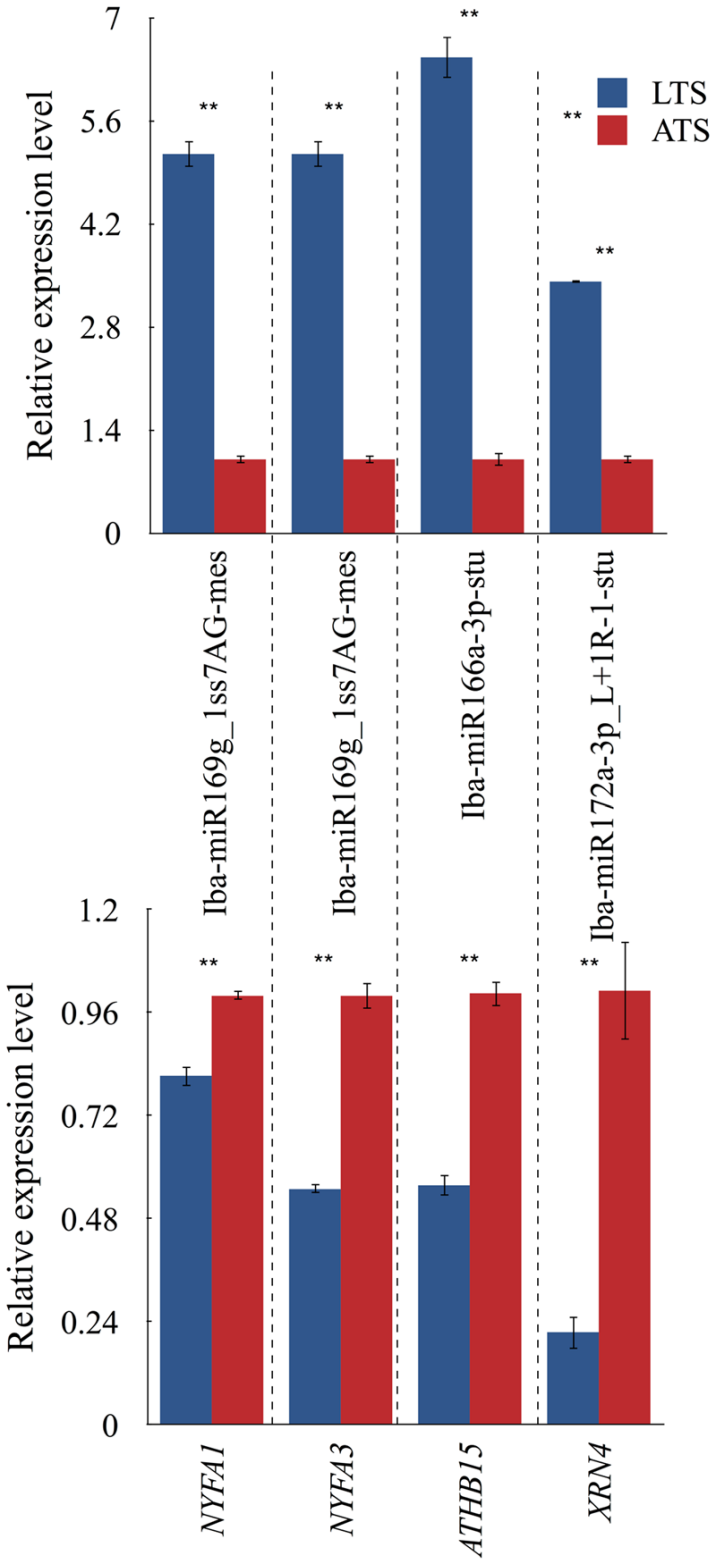
Expression profiles of miRNAs and their target genes under chilling stress by qRT-PCR. Expression of miRNAs and mRNAs was normalized by the level of *U6* and *β-tublin* in qRT-PCR. Three biological replicates were performed for each miRNA and each treatment. An analysis of variance (ANOVA) was employed to analyze all data. The data are presented as the mean ± SD. *p < 0.05, **p < 0.01.

To further validate the relationship between miRNAs and their correspondent targets during chilling treatment, qRT-PCR analysis were performed on three randomly selected miRNAs and their targets (Fig. 7). The negative relationships between the expression of miRNAs (e.g. miR169g_1ss7AG, miR166a-3p_1ss21CA and miR172a-3p_L + 1R-1) and their target genes (e.g. NFYA1 / NFYA3, ATHB-15 and XRN4) was observed; this suggests that miRNAs modulate plant responses to chilling treatment in sweetpotato roots during storage.

## Discussion

### Conserved and novel miRNAs in sweetpotato

To date, a number of *in silico* and wet lab experiments have been performed to identify miRNAs in sweetpotato^13–15^. However, *in silico* identification and characterization of miRNAs and their targets from expressed sequence tags (ESTs), lacks experimental verification and is limited by the number and quality of ESTs. The expression profiles of selected miRNAs during plant growth and development lacks global identification of known and novel miRNAs. The regulation of miRNA and its targets in sweetpotato during storage is still a mystery. Therefore, it is needed to employ multiple omics sources and technologies for systematically analyzing and elucidating the function of miRNAs and their targets in response to chilling stress during sweetpotato storage. In this study, by using deep sequencing technology, we identified a total of 190 known miRNAs in sweetpotato (Table S2); these miRNAs included the common miRNAs that were identified in other plant species. However, miR1508, miR5253, miR5298 and miR2911 were not detected. Since the expression levels of miRNAs differ among different tissues, developmental stages and even genotype- and environmental stress-dependent, this result is not surprising^6^. For example, in a recent study, nearly half of the identified soybean miRNAs were not found in public miRNA database miRBase, likely due to different ecotypes^9^. In this study, 183 different loci were identified which code 191 novel miRNAs. Comparing with the expression of many conserved miRNAs, the expression levels of novel miRNAs were much lower. The results might reflect that the newly risen miRNAs were theoretically more volatile than that in the highly conserved class^22^.

Degradome sequencing has been successfully applied to identify miRNA targets in many plant species^9–11^. In this study, we employed this technology and identified 402 target transcripts targeted by an individual miRNA. These targets play an important role in many plant biological processes, including in plant growth and development as well as plant responses to environmental stresses. Interestingly, 2378 conserved miRNA-target gene pairs and 1200 novel miRNA-target gene pairs were predicted by using psRNATarget. Among the miRNA-target genes pairs, 520 conserved miRNA-target genes pairs and 134 novel miRNA-target genes pairs were supported by the degradome sequencing data. Numerous of candidate miRNA-target genes pairs generated by computational methods were not supported by the degradome sequencing data. One of the potential reasons may be that miRNA regulation also impose translational repression on targets, which would be undetectable by degradome sequencing. Quantitative targeted proteomics approach such as mass spectrometry can be applied generally to validate the remaining candidate miRNA-target genes pairs^23^. Furthermore, the lack of whole genome information may limit the comprehensive identification of miRNAs and their targets, thus further expression analyses in sweetpotato should include more developmental stages to clarify the network between miRNAs and their targets.

### miRNAs response to chilling stress in sweetpotato during storage

Identification of differentially expressed miRNAs could lead to a better understanding of the post-transcriptional regulation taking place in chilling-stressed sweetpotato during storage. Accordingly, we compared the expression of miRNAs between LTS and ATS samples, and classified a total of 26 differentially expressed miRNAs as chilling stress-responsive miRNAs, in which 11 miRNAs targeting mRNAs supported by degradom sequencing. All of these miRNAs were up-regulated in response to chilling stress (Table 1). In other plant species, miR156, miR166, miR169 and miR172 were also found to be significantly responsive to chilling stress^24–28^. In this study, we also found that chilling stress induced the aberrant expression of miR403. This should be the first report on miR403 responsive to chilling stress although there is evidence indicating that miR403 may participate in the developmental regulation of plant^29^. These results indicate that the conserved miRNAs in sweetpotato were similar to other species under chilling stress, while some miRNAs (e.g. miR403) were specific to sweetpotato. Interestingly, all of these miRNAs were up-regulated in response to chilling stress. It is remarkable that miR169 was down-regulated in response to the chilling stress in soybean nodules^30^, and yet can be up-regulated in our study. While these differences could be a result of different target genes and regulatory roles during chilling stress, these patterns of differential regulation could also be due to differences in species or tissue.

### miRNAs play crucial roles in chilling stress by regulating their targets in sweetpotato

**Figure 8:**
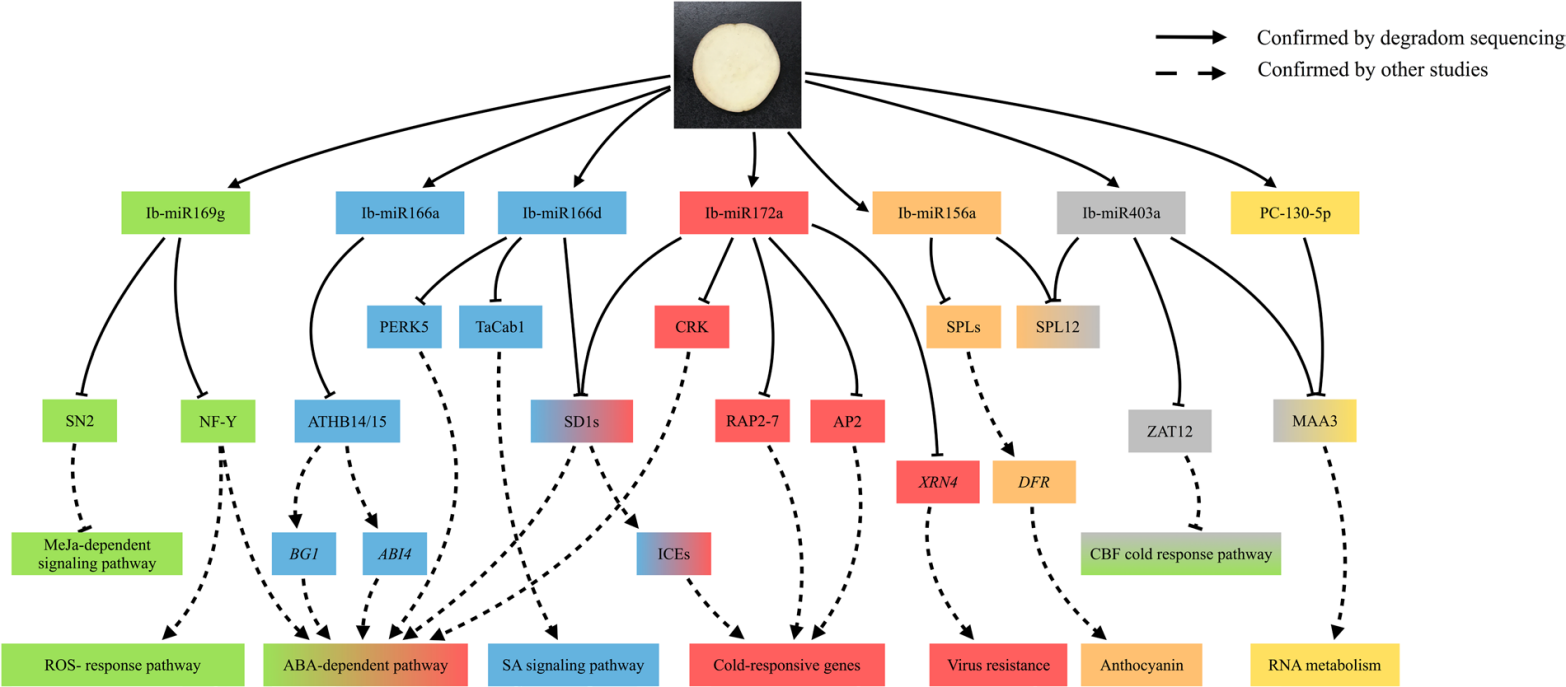
A proposed miRNA-regulatory network in sweetpotato under chilling-stress during storage.

In this study, almost half of chilling responsive miRNAs targeted transcription factors. In addition to transcription factors, we also found that miRNAs target kinase-, helicase-, and protein-coding genes that play roles in adaptive responses to abiotic stress(Fig. 8).

SQUAMOSA PROMOTER BINDING PROTEIN-LIKE (SPL) genes, targeted by miR156, has been predicted in sweetpotato previously^14^, the miR156-*SPL9-DIHYDROFLAVONOL-4-REDUCTASE (DFR)* pathway coordinates the relationship between development and abiotic stress tolerance in plants^31^. Previous study has shown that the expression level of miR156 was significantly lower in storage roots than that in leaves and fibrous roots^13^. This suggests that miR156 may hardly expressed in the tuberous roots. When sweetpotato was exposed to low temperature, the expression of miR156 increased and played a regulatory role. Whether its regulatory mechanism is similar to that of *Arabidopsis* deserves further investigation.

Nuclear factor Y (NF-Y) subunit alpha (NFYA) can inhibit cold stress-induced reactive oxygen species^23^ accumulation and activates abscisic acid^32^-related gene expression^33^, this regulation mechanism was also responsive to abiotic stress in *Arabidopsis* ^34^ and soybean^35^. In this study, NFYA1 and NFYA3 were identified as targets of miR169g. The expression of miR169g was up-regulated by over 21 folds under chilling stress. The expressions of target genes, NYFA1 and NYFA3, showed a significant reduction. The negative relationships between the expression of miR169g and their target genes NYFA1 and NYFA3 suggests that miR169g may regulated NYFA negatively. The decrease of NYFA expression results in the increase of ROS, which may be the reason for the increase of SOD and POD activities. Additionally, miR169 also targets Snakin-2(SN2), a component the methyljasmonate(MeJa)-dependent signaling pathway that is constitutively expressed in plants, although its expression is not usually affected by abiotic stress^36^.

Functions of miR165/166 in plant development and stress responses have been well studied^4^. In A*rabidopsis thaliana*, miR165/166-mediated regulatory module linked to the ABA regulatory network through regulating homeobox-leucine zipper protein (ATHBs) mRNA cleavage, which plays critical roles in drought and cold stress resistance^26^. Five sequences of miR166a, which targeted ATHB-14 and ATHB-15, were identified in this study. As we expected, miR166a were up-regulated whileas ATHB-15 was significantly down-regulated. Moreover, proline-rich receptor-like protein kinase PERK5 and plant receptor-like serine threonine kinase SD1, identified as the targets of miR166, are involved in regulating ABA homeostasis^37^. Therefore, we can hypothesize that the ABA regulatory network which is regulated by miR166 in other species is also present in sweetpotato. Next step, we will examined the expression of ABA-responsive genes, such as *RESPONSIVE TO DESSICATION 29 A (RD29A)*, and core components of ABA signaling pathway, such as ABI1, ABI2 and HAB1 to see how these miRNAs regulate sweetpotato response to chilling stress. Additionally, miR166d also plays a regulatory role in low temperature storage of sweetpotato by targeting EF-hand protein TaCab that is a calcium binding protein accumulating after cold treatment^38^.

Zinc finger protein ZAT12, is a transcription factor acts as a suppressor of CCAAT-binding factor (CBF) transcription factors in response to chilling stress^39^, which is identified as a target of miR403 in this study. Interestingly, miR403 regulated CBF indirectly and miR169 targets NFYA directly, it means CBF transcription factor may play a very important role in response to chilling stress in sweetpotato. Probable helicase MAGATAMA 3 (MAA3), which is also identified as a target of miR403, encodes a homolog of yeast helicase SEN1 that is required for RNA metabolism and may regulate the RNA molecules responsible for nucleolar organization^40^.

In this study, we also found that miR172a targets XRN4, a 5′-3′ exoribonuclease 4 and can enhance viral RNA degradation^41^. The expression of miR172a was up-regulated in LTS whileas XRN4 was down-regulated. This mechanism could be the reason for the decay of sweetpotato by viral infection under low temperature storage condition. In addition, cysteine-rich receptor-like protein kinases (CRK) were also targeted by miR172a, which may control ABA and osmotic stress signaling^42^. Interestingly, PC-130-5p, a novel miRNA identified herein, was only expressed under chilling stress, although expression levels were lower than that of many conserved miRNAs. PC-130-5p potentially targets SEN1^43^ or MAA3^40^. This pathway may be a specific regulatory mechanism in sweetpotato, however, it requires further study.

In this study, several transcription factors, such as *MYB* genes, previously showing that they are responsive to low temperature stress, were not found in sweetpotato roots under chilling stress. Additionally, our *in silico* analysis also identified a set of other transcription factors, such as *WRKY* ^44,45^ and *NAC* ^46^, as the potential miRNA targets in sweetpotato. These genes play an important role in the regulating plant response to chilling stress in other plant species. Thus, these results provide a new clue for future miRNA research on chilling stress during sweetpotato root storage.

In summary, miRNAs and their target genes may co-regulate the MeJa-dependent signaling pathway, ROS-response pathway, ABA-dependent pathway, cold-responsive genes, virus resistance, and anthocyanin and RNA metabolism. Thus, miRNAs regulate a broad number of processes during sweetpotato storage under chilling stress (Fig. 8). sRNAome profiling provides a first look at the miRNA-mediated gene regulation in sweetpotato during chilled storage. Degradome sequencing was employed to identify hundreds of miRNA targets; these targets reveals the complicated interactions between miRNAs and their targets in sweetpotato. Although the complex miRNA-mediated regulatory networks are still somewhat in a mystery, this dataset represents an important supplement to the existing sweetpotato miRNA database, and should be useful in understanding the complex gene regulatory networks in sweetpotato. Additionally, 26 chilling-responsive miRNAs were identified, thereby providing a foundation for the regulatory function of miRNAs in sweetpotato under chilling stress.

## Methods and Materials

### Plant materials, sample collection and RNA isolation

The experiments were performed in the School of Life Science, Jiangsu Normal University (Xuzhou, China). A commonly used and chilling-sensitive sweetpotato (*Impomoea batatas L*.) cultivar ‘Xushu 18′ was grown in the Key Laboratory of Biology and Genetic Improvement of Sweetpotato of Ministry of Agriculture, Xuzhou. After harvest at the same stage of maturity, unblemished and disease-free tuberous roots with similar size and shape were selected. To minimize microbial effects, all samples were dipped in 500 mg/L thiabendazole (Jiangsu Xuzhou Shennnong Chemicals Co.,Ltd.) for 3 minutes and air dried. Cleaned tuberous roots were healed at 35 °C with a relative humidity of 85% for 3 days, then divided into two groups. One group was stored at 14 °C for appropriate temperature storage^42^; another group was stored at 4 °C for low temperature storage^47^, both with a relative humidity of 85%. Tuberous roots were collected from each group each week for 5 weeks, and immediately frozen in liquid nitrogen and stored at −80 °C freezer. We pooled samples together to eliminate effects of individual genetic variance. Tissues from seven tuberous roots were mixed as one biological replicate. In total, three independent biological replicates were collected for each treatment at each time point. Total RNAs were extracted using Trizol (Invitrogen, CA, USA). The quantity and purity of total RNA were analyzed with a Bioanalyzer 2100 and RNA 6000 Nano LabChip Kit (Agilent, CA, USA) with RIN number > 7.0. Samples with 1–5 weeks of treatments for ATS and LTS were used for measuring the contents of MDA, starch and sucrose, and the activities of POD and SOD. Samples with 4 weeks of treatment were used for sequencing and qRT-PCR analysis. Based on the test on physiological responses, we found that 4 weeks of chilling treatment caused significantly changes; thus, we detected the miRNA expression in the samples with 4 weeks of chilling treatment.

### Construction, sequencing, and analysis of small RNA libraries

Small RNA libraries were constructed using about 1 ug of total RNAs from the 4 weeks of ATS and LTS treatments according to the protocol of TruSeq Small RNA Sample Prep Kits (Illumina, San Diego, USA). Single-end sequencing was performed on an Illumina Hiseq. 2500 by the LC-BIO (Hangzhou, China).

Identification of known and novel miRNAs in sweetpotato tuberous roots was performed according to a reported approach with a minor modification^48^. Briefly, all raw reads were filtered with the Illumina pipeline filter, and then processed with the ACGT101-miR program (LC Sciences, Houston, Texas, USA) to remove adapter dimers, low-quality, low complexity and low copy reads; the common RNA families, rRNA, tRNA, snRNA, snoRNA, were also removed during this stage. Then, the rest clean small RNA sequences with 18–25 nt in length were compared with the public miRNA database, miRBase 21 using BLASTn to identify known miRNAs. Then, the unmapped sequences were subjected to blast against the sweetpotato genome from Kazusa DNA Res. Inst.^20^ (http://ftp.kazusa.or.jp/pub/sweetpotato/0431-1/). The genome and assembled unigenes to identify novel miRNAs and predicated stem-loop hairpin secondary structures by using RNAfold software (http://rna.tbi.univie.ac.at/ cgi-bin/RNAfold.cgi). The criteria for identifying novel miRNAs was followed a previous report^48^. Novel miRNAs were only considered when the MFEIs of their pre-miRNAs were ≥ 0.80^21^. Moreover, the normalized copy number of the novel miRNAs was required to be ≥ 10 in at least one small RNA library to avoid potential false positives.

### Degradome library construction and target identification

A degradome library was constructed from 20 ug of sweetpotato total RNAs and sequenced on an Illumina Hiseq. 2500 to identify mRNA targets following a previous method^49^. Raw sequencing reads were obtained using Illumina’s Pipeline v1.5 software followed by sequencing image analysis with Pipeline Firecrest Module and base-calling by Pipeline Bustard Module. A public software package, CleaveLand3.0^50^ was used for analyzing sweetpotato miRNA targets. All identified targets were subjected to a BlastX analysis to search for similarity. A GO analysis was employed to uncover the miRNA-gene regulatory network on the biological process and molecular function. GO enrichment analysis was conducted with hypergeometric distribution (LC-BIO, Hangzhou, China). The formula for calculation is

$$P=1-\sum _{i=0}^{S-1}\frac{(\begin{array}{c}B\\ i\end{array})(\begin{array}{c}TB-B\\ TS-i\end{array})}{(\begin{array}{c}TB\\ TS\end{array})}$$

where TB was total number of mRNAs; TS was the number of mRNAs corresponding to selected miRNAs; B was the number of mRNAs annotated with specific functions; S was the number of mRNAs annotated with specific functions corresponding to selected miRNAs; *P* value ≤ 0.05 was regarded as the threshold.

### Expression pattern of chilling stress-responsive miRNAs

To investigate the differentially expressed miRNAs responsive to chilling stress in sweetpotato, miRNAs read count was normalized to the total number of miRNA tags per million (TPM) to correct copy numbers among different libraries. Samples from LTS were compared with ATS to investigate the differentially expressed miRNAs responsive to chilling stress in sweetpotato during root storage. The differential expression was based on normalized deep-sequencing counts using an ANOVA. The *P* value of the Students t test was ≤ 0.05, absolute value of |log2ratio| was ≥ 1, indicating the ratio of TPM values for the LTS and ATS libraries. When it fits to these criteria, we considered this miRNA was significant on the changes of gene expression.

### Validation of miRNAs and their targets

To confirm the expression of the identified miRNAs and their correspondent targets, we randomly selected miRNAs and their target genes with different expression levels for qRT-PCR. The analysis was performed using samples with 4 weeks of treatments from ATS and LTS. The miRNA qRT-PCR reactions were performed by LightCycler^®^ 96 (Roche, Switzerland) using Mir-X™ miRNA qRT-PCR SYBR^®^ Kit (Takara, China) in triplicate for each sample. The qRT-PCR temperature program as following: 95 °C for 10 min followed by 40 cycles of 95 °C for 5 sec, and 60 °C for one min. During miRNA expression analysis, snRNA *U6* was served as reference gene (Table S8). During the validation of target genes, *β-tublin* was served as reference gene and the primer sequences were listed in the supplementary documents (Table S9). The qRT-PCR reaction was performed in LightCycler^®^ 96 (Roche, Switzerland) using One Step SYBR^®^ PrimeScript^TM^ RT-PCR Kit II (Takara, China) in three independent biological replicates. The PCR was programmed as follows:42 °C for 5 min; 95 °C for 10 sec; 40 cycles of 95 °C for 5 sec; 60 °C for 40 sec; and 95 °C for 10 sec; 65 °C for 15 sec; 95 °C for 10 sec. 2^−∆∆CT^ method was employed to analyze the relative changes of both miRNA and protein-coding genes^51^. An analysis of variance (ANOVA) was employed to analyze all data generated from qRT-PCR.

## Electronic supplementary material

Supplemental Figures

Dataset 1-9
