# Supplementary material for: High throughput deep sequencing reveals the important roles of microRNAs during sweetpotato storage at chilling temperature
Source: Sci Rep. 2017 Nov 29;7:16578. doi: 10.1038/s41598-017-16871-8 (PMC5707365; doi:10.1038/s41598-017-16871-8)
Supplement: Supplementary file 1 — Supplemental Figures [file 41598_2017_16871_MOESM1_ESM.pdf]

*Supplementary Material*

**to**

# High throughput deep sequencing reveals the important roles of microRNAs during sweetpotato storage at chilling temperature

**Zeyi Xie[1, 2, &], Aiming Wang[1, 2, &], Hongmin Li[3, 4], Jingjing Yu[1, 2], Jiaojiao Jiang[1, 2], Zhonghou Tang[3, 4], Daifu Ma[3, 4], Baohong Zhang[5], Yonghua Han[1, 2*] & Zongyun Li[1, 2*]**

[1]Institute of Integrative Plant Biology, School of Life Science, Jiangsu Normal University, Xuzhou, China

[2]Jiangsu Key Laboratory of Phylogenomics and Comparative Genomics, Jiangsu Normal University, Xuzhou, China

[3]Xuzhou Institute of Agricultural Sciences in Xuhuai Distrct, Jiangsu Xuzhou Sweetpotato Research Center, Sweetpotato Research Institute, CAAS, China

[4]Key Laboratory of Biology and Genetic Improvement of Sweetpotato, Ministry of Agriculture, Xuzhou, China

[5]Department of Biology, East Carolina University, Greenville, NC, USA

&: co-1st author

*** Correspondence:**

Zongyun Li and Yonghua Han

zongyunli@jsnu.edu.cn (ZL) and hanyonghua@jsnu.edu.cn (YH)

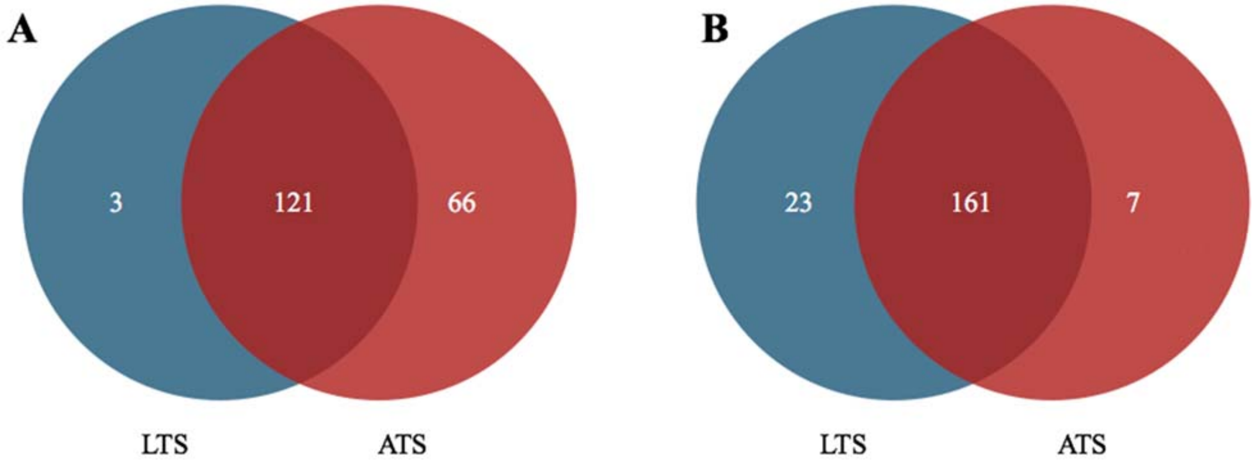


**Supplementary Figure 1.** Venn diagram showing the numbers of known miRNA and novel miRNAs found for NS, LTS and ATS sweet potatoes**.**

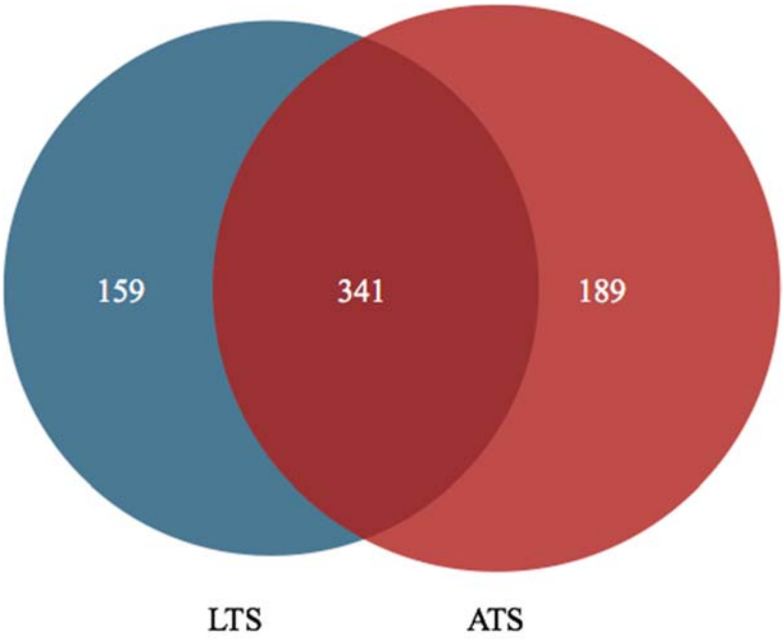


**Supplementary Figure 2.** A Venn diagram showing the numbers of miRNA-target gene pairs found for NS, LTS and ATS p sweet potatoes.

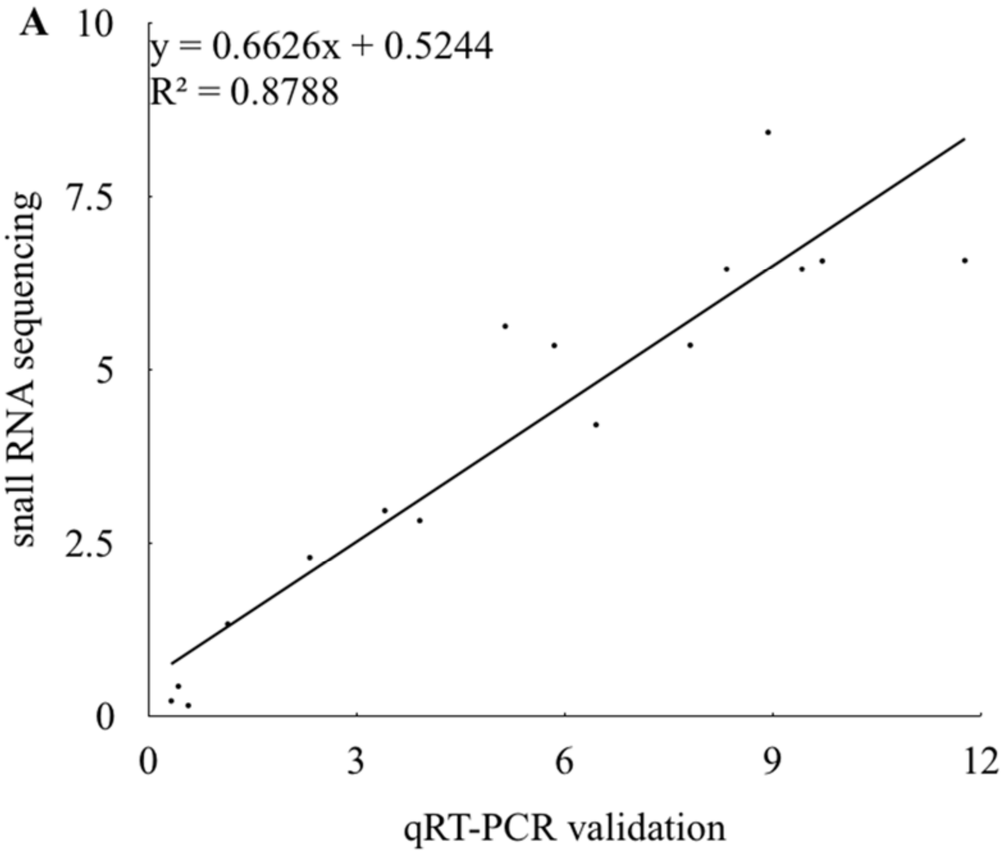


**Supplementary Figure 3.** Coefficient analysis between data obtained from sequencing and qRT-PCR for miRNA expression.
